# HIV detection by an emergency department HIV screening program during a regional outbreak among people who inject drugs

**DOI:** 10.1371/journal.pone.0251756

**Published:** 2021-05-18

**Authors:** Kiran A. Faryar, Rachel M. Ancona, Zachary Reau, Sheryl B. Lyss, Robert S. Braun, Todd Rademaker, Ryane K. Sickles, Michael S. Lyons

**Affiliations:** 1 Department of Emergency Medicine, University of Cincinnati Medical Center, Cincinnati, OH, United States of America; 2 HIV, STI, and Viral Hepatitis Interventions and Treatment Section, Office of Health Improvement and Wellness, Ohio Department of Health, Columbus, OH, United States of America; 3 Division of HIV/AIDS Prevention, Centers for Disease Control and Prevention (CDC), Atlanta, GA, United States of America; 4 HIV Testing, Care, and Prevention, Hamilton County Public Health, Cincinnati, OH, United States of America; University of Arkansas for Medical Sciences, UNITED STATES

## Abstract

**Objective:**

Multiple HIV outbreaks among persons who inject drugs (PWID) have occurred in the US since 2015. Emergency departments (EDs), recognized as essential venues for HIV screening, may play a unique role in identifying undiagnosed HIV among PWID, who frequently present for complications of injection drug use (IDU). Our objective was to describe changes in HIV diagnoses among PWID detected by an ED HIV screening program and estimate the program’s contribution to HIV diagnoses among PWID county-wide during the emergence of a regional HIV outbreak.

**Methods:**

This was a retrospective study of electronically queried clinical records from an urban, safety-net ED’s HIV screening program and publicly available HIV surveillance data for its surrounding county, Hamilton County, Ohio. Outcomes included the change in number of HIV diagnoses and the ED’s contribution to case identification county-wide, overall and for PWID during 2014–2018.

**Results:**

During 2014–2018, the annual number of HIV diagnoses made by the ED program increased from 20 to 42 overall, and from 1 to 18 for PWID. We estimated that the ED contributed 18% of HIV diagnoses in the county and 22% of diagnoses among PWID.

**Conclusions:**

The ED program contributed 1 in 5 new HIV diagnoses among PWID county-wide, further illustrating the importance of ED HIV screening programs in identifying undiagnosed HIV infections. In areas experiencing increasing IDU, HIV screening in EDs can provide an early indication of increasing HIV diagnoses among PWID and can substantially contribute to case-finding during an HIV outbreak.

## Introduction

HIV remains a high-priority health threat, and improved diagnosis of HIV represents one of the four pillars in the national Ending the HIV Epidemic initiative [1]. Screening for undiagnosed HIV infection is critical to stemming the epidemic. Approximately 30% of HIV transmissions occur from persons with undiagnosed infection [2], and the benefits of earlier diagnosis for reduced transmission [3] and improved health outcomes [3] are well-understood [4–6]. Nationally, during 2014–2018, the total number of HIV diagnoses declined but the number of diagnoses attributed to injection drug use (IDU) increased [7]. HIV epidemiology can vary over time, both geographically and by mode of transmission. HIV outbreaks among persons who inject drugs (PWID) have occurred in multiple regions in locations such as Scott County, Indiana [8], Seattle, Washington [9]; Northeastern Massachusetts [10]; Philadelphia, Pennsylvania [11]; and Cabell County, West Virginia [12].

During 2017–2018, an HIV outbreak among PWID was identified in the Cincinnati/Northern Kentucky area, including Hamilton County, the location of the current investigation [13]. As the injection of opioids and other substances continues nationally [14], there is a critical need to screen PWID as a priority population to enable early detection of emerging HIV outbreaks.

Emergency departments (EDs) have been emphasized as essential venues for HIV screening [4, 15]. Typically, large patient volumes [16], 24/7 operation [17], and availability of treatment regardless of ability to pay [18] result in ED access to significant numbers of persons with undiagnosed HIV [19]. Expanding the availability of HIV testing in EDs is a recommended activity to support the diagnosis of all individuals with HIV as early as possible after infection as part of the federal Ending the HIV Epidemic initiative. PWID present to EDs at a higher rate than the general population and for reasons often associated with IDU, such as overdose and injection-related infections [20, 21]. In 2017, there were nearly one million ED visits for non-fatal overdose in the United States [22]. Because EDs have ready access to the PWID population, they may be uniquely poised to identify PWID with undiagnosed HIV.

The goal of this investigation is to describe changes in HIV diagnoses among PWID by an ED HIV screening program in Hamilton County, Ohio, and estimate the program’s contribution to diagnosing cases of HIV among PWID county-wide during the emergence of a regional HIV outbreak.

## Materials and methods

### Study design and setting

We performed a retrospective study of i) electronically queried clinical record data from an urban, academic ED that serves as the region’s primary source of episodic, unscheduled safety-net care, and ii) publicly available aggregated surveillance data from Hamilton County, Ohio[23], where the ED is located. This study was approved by the University of Cincinnati academic Institutional Review Board with a waiver of informed consent.

In 2017, an HIV outbreak among PWID was identified in the Cincinnati metro area; the majority of diagnoses occurred in Hamilton County. Hamilton County has had the highest rate of new HIV diagnoses (17.4–22.9 per 100,000 population) in Ohio since 2014 [24], and Ohio had the fourth highest rate of opioid-related overdose deaths (29.6 per 100,000 population) in the country in 2018 [25]. The study-site ED, University of Cincinnati Medical Center, has approximately 75,000 visits annually and does not operate affiliated urgent care centers. Hamilton County is the third most populous county in Ohio with a population of 813,589 [26]. To our knowledge, none of the other fourteen EDs in the county are engaged in any sizeable or systematic HIV screening efforts. Although this academic center does encounter patients from surrounding counties, this percent, as well as the percent of HIV diagnoses from residents of surrounding counties, is small in comparison to Hamilton County residents. We present our results in the context of public health surveillance reports which are tabulated by county of residence rather than county of diagnosis.

Although diagnosis codes are known to misclassify and severely underestimate ED prevalence of substance use disorders [27–29], we characterized the study-site ED population from 2017–2018 using International Classification of Diseases 10^th^ Edition (ICD-10) codes. Of ED patients, 0.7% had at least one opioid use disorder diagnosis (any F19.X), 0.3% had a diagnosis of intravenous drug use (F19.9), 1% had either a chief complaint or diagnosis code that included the terms “opioid” and “overdose”, and 2% had either a chief complaint or diagnosis code that included the term “abscess” [21].

### ED HIV screening program and local health department

This ED has operated a publicly funded HIV screening program since 1998 [30]. More recently, screening is additionally supported by Gilead Sciences’ FOCUS program and by conventional healthcare financing of usual clinical operations in the ED. The screening program has four active modes (approaches or reasons for testing) [5, 30]: 1) non-targeted opt-out screening of all persons aged <65 years, driven by prompts to action in triage workflows of the electronic health record (EHR) system which were active from June 23—December 29, 2015 and April 1, 2017—December 31, 2018, 2) risk-targeted HIV screening led by a parallel program operated by publicly funded adjunct health professionals, 3) “walk-in” clients who come to the ED seeking HIV testing and are served by the program’s staff but are not requesting ED care nor registered as ED patients, and 4) targeted (or diagnostic) testing of patients whom providers identify with clinical signs or symptoms of HIV or risks for HIV acquisition.

These four modes employ two testing processes. For ED patients who are risk-targeted or “walk-in” clients (modes 2 and 3), HIV screening and confirmation are conducted through a rapid/rapid testing algorithm: OraQuick *ADVANCE*® HIV-1/2 Rapid Antibody Test (OraSure Technologies, Bethlehem, PA) followed by the INSTI® HIV-1/HIV-2 Antibody Test (bioLytical Laboratories, Inc., Richmond, BC, Canada) if results on the OraQuick are reactive. For non-targeted opt-out screening and diagnostic tests (modes 1 and 4), an HIV antigen/antibody (Ag/Ab) test (ARCHITECT® HIV Ag/Ab Combo, Abbott Laboratories, Abbott Park, IL) is used as the screening assay. Reactive results are followed by a type-differentiating assay (Geenius HIV-1/2 Supplemental Assay, Bio-Rad Laboratories, Inc., Hercules, CA). If results of the type-differentiating assay are indeterminate, a qualitative HIV-1 ribonucleic acid (RNA) assay will be used. The Ag/Ab test has been in use since January 2017; during 2014–2016, an immunoassay that detected IgG/IgM only was used as the screening assay. All screening approaches follow both Ohio Department of Health [31] and CDC [32] recommended algorithms for confirmatory diagnosis where diagnosis can be confirmed by double positive rapid assay (rapid/rapid) or combined antibody differentiation immunoassay.

Patients undergo a comprehensive risk assessment in conjunction with the screening program’s result notification and linkage to care process. If staff is unable to interview a patient (either cannot make contact or patient declines), they will perform a structured chart review of the EHR for indications of HIV behavioral risk factors.

Testing program staff use records review, patient interview, and collaboration with the local health department to determine which individuals are newly versus previously diagnosed. The testing program also works to ensure persons with new or repeat HIV diagnoses are linked to medical care and social services, as needed. When HIV is diagnosed among ED patients, diagnoses are reported to the state health department within 24 hours. The local health department designates a diagnosis as new by checking the Ohio Disease Reporting System for a prior diagnosis, requesting prior test history from the test site (prior results, either from medical record or self-reported by the patient), and discussion with the individual as part of the disease investigation process. If there is evidence of the individual residing previously in another state, the local health department will also perform state-to-state record requests through the Ohio Department of Health.

The local health department and our ED consider both permanent residents in the county and persons without a permanent residence (i.e., unstably housed) who were tested at our site to be Hamilton County residents.

### Study data acquisition and measurements

We performed an electronic query of ED’s HIV screening program data (2014–2018) to define the study sample and patient characteristics. We included all persons who: 1) had a confirmed result on the HIV testing algorithm [32], 2) had an HIV diagnosis classified as “new” by testing program and local health department procedures, and 3) were Hamilton County residents as defined by the testing program and local health department. Additional patient characteristics electronically queried from the ED EHR included the following ED chief complaint and discharge diagnoses: opioid use disorder [33], drug overdose [27], bacterial infection [34], and hepatitis C [35]. These have been identified as i) evidence-based medical conditions experienced by PWID at a disproportionately higher rate [27, 33–35], and ii) medical conditions reported as missed opportunities for ED HIV testing in a recent publication [13], specifically for the regional PWID HIV outbreak discussed in this paper.

Opioid use disorder (OUD) was defined as any OUD diagnosis (any diagnosis that included the terms: “opioid use”, “opioid dependence”, or “opioid use disorder”). Drug overdose was defined as either a chief complaint or discharge diagnosis containing the term “overdose”. Bacterial infections included any chief complaints or diagnoses with the following terms: “abscess”, “bacteremia” or “other bacterial infection”, “cellulitis”, “disorder of skin and subcutaneous tissue”, “osteomyelitis”, “sepsis” or “septic arterial embolism”, or “staphylococcus”. Hepatitis C diagnoses only included those who were tested at their HIV diagnostic visit (diagnosis included term “hepatitis C”).

We acquired aggregate data on the number of HIV diagnoses among Hamilton County residents and risks for HIV acquisition (i.e., male to male sexual contact, injection drug use, high-risk heterosexual behavior) from publicly available Ohio Department of Health reports. Individual-level identifiable data county-wide was not available; therefore ED patient-level data was not matched one-to-one with county data. For both ED and county-wide data, we classified both persons with IDU only and those with both male-to-male sexual contact and IDU as PWID.

We additionally identified PWID who experienced homelessness by electronically querying the ED HIV screening program’s data (homeless documented by program staff during pre-test or post-test interview) and the EHR (diagnosis that included the term “homelessness”). Homelessness is not only associated with higher rates and greater severity of substance use disorders, including opioid use disorder and opioid overdose in ED populations, but also (along with hepatitis C) highly prevalent in reports of HIV PWID outbreaks nationwide, including Seattle [9] and Massachusetts [10].

### Analysis

The analysis for this study was descriptive. Our primary outcomes were the number of persons with HIV newly diagnosed by the ED’s screening program in each year of the study period and the ED’s estimated contribution (proportion) to identification of new HIV diagnoses county-wide, overall and for PWID. Proportions for primary outcomes were reported with their respective 95% confidence intervals (CIs). We secondarily detail characteristics of PWID (demographics, chief complaints and diagnoses associated with IDU, and homelessness) at the time of diagnosis. Patients with missing data were retained and noted where applicable. Analyses were completed in R (version 3.4.1.; R Foundation for Statistical Computing) [36].

## Results

During the five-year study period, 142 persons received new HIV diagnoses through the ED HIV screening program. Persons with new HIV diagnoses were primarily black/African American (93, 65.5%), non-Hispanic/Latino (139, 97.9%), and male (109, 76.8%) with a median age of 31 years. Fourteen (9.9%) did not have an identified risk factor for HIV acquisition. Thirty-seven (26%) new HIV cases were among PWID, including two who had the combined acquisition risk of male-to-male sexual contact and IDU. The 37 PWID with newly diagnosed HIV at the study-site ED were primarily white (30, 81.1%), non-Hispanic/Latino (37, 100%), and male (25, 67.6%) with a median age of 33 years (Table 1).

**Table 1:** Persons newly diagnosed with HIV at study-site ED from 2014–2018, overall and stratified by Injection Drug Use (IDU) classification.

	Total	IDU	No IDU*
	(N = 142)	(N = 37)	(N = 105)
**Age**^^^	31 (24–40)	33 (28–37)	30 (23–41)
**Gender**			
Male	109 (76.8)	25 (67.6)	84 (80.0)
Female	29 (20.4)	12 (32.4)	17 (16.2)
Transgender (MTF)	4 (2.8)	0 (0.0)	4 (3.8)
**Ethnicity**			
Not Hispanic/Latino	139 (97.9)	37 (100)	102 (97.1)
Hispanic/Latino	3 (2.1)	0 (0.0)	3 (2.9)
**Race**			
Black/African American	93 (65.5)	7 (18.9)	86 (81.9)
White/Caucasian	44 (31.0)	30 (81.1)	14 (13.3)
Asian	1 (0.7)	0 (0.0)	1 (1.0)
Multiracial/Biracial	1 (0.7)	0 (0.0)	1 (1.0)
Other	3 (2.1)	0 (0.0)	3 (2.9)
**Risks for HIV Acquisition**			
Male to male sexual contact	48 (33.8)	2 (5.4)	46 (43.8)
Injection drug use (IDU)	37 (26.1)	37 (100)	0 (0.0)
High-risk heterosexual behavior	73 (51.4)	16 (43.2)	57 (54.3)

* This includes 11 ED patients whose IDU status was unknown.

^**^**^ All columns are reported as numbers with proportions, except for age, which is reported as median and interquartile range.

Over the study period, 142/808 (17.6%, 95%CI 15.0–20.4) of all HIV diagnoses county-wide and 37/165 (22.4%, 95%CI 16.5–29.7) of diagnoses among PWID were made in the study-site ED. Annual testing numbers and study-site ED contribution for newly diagnosed individuals with and without IDU are shown in Table 2. From 2014 to 2018, the annual number of persons with HIV newly diagnosed through the ED screening program increased from 20 to 42, and the number and percent of diagnoses involving PWID increased from 1 (5.0%) to 18 (42.9%). The ED’s contribution to HIV diagnoses in the county increased over time from 13.2% to 22.7%.

**Table 2:** Persons newly diagnosed with HIV with and without injection drug use by year and location.

	ED*		Hamilton County^		ED Contribution	
	Total	PWID*	Total	PWID*	Total	PWID*
Year	N	N (%)	N	N (%)	%	%
2014	20	1 (5.0)	152	15 (9.9)	13.2	6.7
2015	23	2 (8.7)	144	3 (2.1)	16.0	66.7
2016	23	4 (17.4)	141	16 (11.3)	16.3	25.0
2017	34	12 (35.3)	186	42 (22.6)	18.3	28.6
2018	42	18 (42.9)	185	65 (35.1)	22.7	27.7
**Total**	**142**	**37 (26.1)**	**808**	**165 (20.4)**	**17.6**	**22.4**

* ED = emergency department; PWID = persons who inject drugs.

^**^**^ Includes emergency department diagnoses from study site ED.

The number of all individuals with new HIV diagnoses and PWID with new HIV diagnoses by each screening modality in use by the ED program are shown in Table 3. Table 4 illustrates that among newly diagnosed ED patients with IDU, 28 (75.7%) had at least one IDU-associated condition that was identified at the time of their diagnostic ED visit.

**Table 3:** ED patients newly diagnosed with HIV by screening modality and Injection Drug Use (IDU) classification.

Screening Modality*	Total (N = 142)	IDU (N = 37)	No IDU (N = 94)	IDU Unknown (N = 11)
Non-targeted opt-out	21 (14.8)	9 (24.3)	9 (9.6)	3 (27.3)
Risk-targeted	52 (36.6)	11 (29.7)	39 (41.5)	2 (18.2)
Walk-in clients	3 (2.1)	0 (0.0)	3 (3.2)	0 (0.0)
Diagnostic tests	66 (46.5)	17 (45.9)	43 (45.7)	6 (54.5)

* All columns are reported as numbers with proportions.

**Table 4:** High-risk medical conditions seen in ED patients newly diagnosed with HIV and reporting injection drug use [13, 27, 33–35].

	N* (%)
**Accidental overdose**	7 (18.9)
**Bacterial infection**	12 (32.4)
Abscess	9 (24.3)
Cellulitis	6 (16.2)
Bacteremia	4 (10.8)
Sepsis	4 (10.8)
Staphylococcus	4 (10.8)
Disorder of skin/tissue	1 (2.7)
Other bacterial infection	1 (2.7)
**Hepatitis C**	13 (35.1)
**Homelessness**	18 (48.6)

* N = 37.

## Discussion

Hamilton County, Ohio has experienced an outbreak of HIV among PWID. Given the growing number of recent HIV outbreaks among PWID reported across the U.S. [8–13, 37–39], collective understanding must shift from awareness that outbreaks can happen to awareness that outbreaks are happening, presumably with some outbreaks currently ongoing but not yet identified and others likely to develop in the future. This analysis reports the contribution of a single, large-volume urban ED screening program to identification of new HIV cases, particularly among PWID, and its contribution to local and state public health department surveillance during the emergence of this regional outbreak. The finding that a single, large-scale ED screening program identified 1 in 5 cases of HIV among PWID for the entire county calls for an expanded view of the potential role of EDs in the identification of previously undiagnosed HIV, detection of trends including outbreaks, and even outbreak response.

The importance of ED case identification and linkage to care to improve individual health outcomes and reduce the chance of further transmission is well-supported by existing literature and recommended by public health authorities [4, 15, 40]. The importance of the ED’s contribution to detecting HIV outbreaks among PWID, however, has only recently been realized. During a recent outbreak among PWID in Philadelphia, many of the new diagnoses were made in EDs [11]. The idea of EDs playing a role in syndromic surveillance is not novel and has been reported previously for other non-HIV infections, including Ebola, COVID-19-like illness (CLI) or influenza-like illness (ILI) [41, 42]. Because of enhanced access to priority and often hidden populations, it is theoretically possible that EDs could play a role as the proverbial “canary in the coal mine” by identifying changes in HIV case identification and trends. One could posit that ED screening could have unique value for surveillance of potential future changes, even if there is little current value in the screening because of minimal numbers of new diagnoses.

The best method, or combination of methods, by which EDs could play an expanded role in supplying data to surveillance systems is not fully elucidated. The potential contribution of screening programs is illustrated by this report. It might also be possible to expand large-scale and automated syndromic surveillance approaches. For the example of injection drug use, algorithms could be programmed to identify chief complaint and diagnosis criteria such as described in this report. Expanded screening, public health investigation, or other measures could be triggered by passing a certain threshold in the frequency of those conditions. Also of interest is the degree to which other episodic, unscheduled care centers might contribute, such as urgent care settings and stand-alone EDs.

Our retrospective analysis revealed that the increase in the number of PWID with newly diagnosed HIV in this ED paralleled county-wide increases. This suggests that EDs could potentially be a special focus for public health surveillance entities to identify trends earlier by evaluating ED data in real time. However, this must be interpreted with caution. The change in the characteristics of persons with newly diagnosed HIV in our ED screening program was not recognized prospectively, and there is no certainty that having done so would have constituted an earlier detection of this trend. Even in retrospect, drastic increases in the proportion of HIV diagnoses among PWID in our ED during 2014–2016 from small numbers, e.g., from 1 (5.0%) in 2014 to 4 (17.4%) in 2016, could have simply been due to random variation. Not until 12 (35.3%) diagnoses were made in 2017 was the change in pattern an obvious one. This suggests that interpreting such data in the context of other factors–such as the known increase in unintentional overdoses in the area long before the HIV outbreak occurred–is particularly critical when small increases in the absolute number of diagnoses are observed. While it is interesting to consider that ED access to key populations may have broad relevance in sentinel surveillance (e.g. if an HIV outbreak were to occur among men with male-to-male sexual contact), we do not know the degree to which the findings of this report are specific to HIV or PWID.

Regardless of whether or not the ED can play a role in sentinel detection of an HIV outbreak, these findings demonstrate the ED’s potential for contributing to case finding during an outbreak. The existence of our multi-modal HIV screening program prior to the outbreak allowed for identification of increased numbers of HIV diagnoses at the onset of the regional outbreak. We recognize that this was only one ED with a multi-modal, but not fully comprehensive, screening program. In fact, our ED and local health department were well aware of the increase in IDU and unintentional overdose long before the HIV outbreak. Perhaps implementation of programmatic changes such as testing all patients who present to the ED with an injection-related infection (e.g. abscess) or non-fatal overdose would have led to earlier outbreak detection, once EDs were aware of the regional IDU increase. We are aware that there were missed opportunities for HIV testing among PWID in our ED who were later diagnosed during the outbreak [13] using our existing multi-modal screening approach.

The majority of PWID receiving HIV diagnosis had at least one condition associated with IDU identifiable in the EHR at the time of their HIV diagnosis. This finding implies that EDs, particularly those in areas with a high prevalence of IDU, should prioritize HIV testing for all patients with conditions associated with IDU even if not otherwise engaged in recommended large-scale screening efforts. Educating physicians about the need for increased testing in patients presenting with such conditions (which was echoed in a recent CDC Health Advisory [38]), EHR modifications to auto-order a test in systems where that is possible, or auto-alerts prompting an HIV test when these chief complaints or diagnoses are present in patient records could facilitate HIV screening in low-resource, high-IDU areas. Our ED screening program educated our emergency providers on the regional HIV outbreak to prompt increased HIV testing among patients with IDU-associated conditions, program staff have placed renewed emphasis on screening patients presenting with IDU-associated conditions, and program leadership is working to refine EHR prompts.

This study has several limitations, the first of which is generalizability. The number of positive HIV diagnoses identified by an ED and the overall contribution of those diagnoses to surveillance will vary by ED, however the overall process by which EDs might contribute to surveillance through information transfer can be generalizable across regions. HIV diagnoses classified as new by our program and the local health department might have subsequently been determined at the state or national level to have been previously diagnosed in another jurisdiction. However, this is unlikely to have a large influence on i) the number of diagnoses among PWID from our ED or ii) the increasing number of diagnoses that occurred among PWID in our ED during this time period. We may have underestimated or misclassified the number of diagnoses among PWID if patients did not complete a risk assessment interview and IDU was not documented in the EHR. Further surveillance efforts to identify missing information about risks for HIV acquisition would only have increased the number of cases attributed to IDU. We have likely underestimated the proportion of PWID with hepatitis C exposure since not all PWID were tested, and we did not assess prior hepatitis C diagnoses in the EHR. We may have also underestimated the proportion of IDU by excluding stimulant or other substance use disorders. Anecdotally, polysubstance use and stimulant use were less prevalent during the 2017 outbreak than it is currently. Any inclusion of polysubstance use, other substance use disorders, or stimulant related IDU would have only increased the number of HIV diagnoses identified. It is also possible that some of the individuals with HIV diagnosed by the ED screening program classified as PWID were not actively using injection drugs at the time of infection, or that PWID may have still acquired HIV through sexual contact. ED screening program changes may have accounted for part of the increase in number of diagnoses among PWID, particularly the increase in non-targeted opt-out screening with the implementation of EHR-facilitated screening in triage (starting in April 2017). Even given these limitations, it does not diminish the finding that our ED screening program was able to detect a relatively large number of newly diagnosed HIV cases among PWID in the county.

## Conclusions

In summary, EDs naturally encounter patients who may not otherwise seek healthcare, including those who are seeking care specifically because of an acute condition as well as patients with less acute or chronic conditions who cannot or do not choose to go elsewhere. This juxtaposition of being a care source for both acute symptoms and disadvantaged populations is the likely explanation for the finding that EDs are uniquely poised to diagnose HIV among PWID and, for that reason, play a key role in local plans to End the HIV Epidemic. ED HIV screening, already recognized for the direct benefit of HIV diagnosis to individual clinical outcomes, should also be valued for the data contributed to detection of outbreaks or other changing trends. ED HIV screening programs operating at large-scale and with access to hidden populations can help detect changes in HIV transmission before or during the emergence of a regional HIV outbreak. In areas experiencing increased rates of IDU or other circumstances that would render the community vulnerable to sudden or unrecognized increases in HIV infection, HIV screening in EDs can provide an early indication of an increase in HIV diagnoses among PWID in a community. Should an HIV outbreak among PWID occur, HIV screening programs can substantially contribute to case finding and, more broadly, to outbreak response.
